# Co-existing Paget's disease and ankylosing spondylitis resulting in panthoracic pagetic vertebral ankylosis

**DOI:** 10.1259/bjrcr.20150405

**Published:** 2016-05-28

**Authors:** David McKean, Alpesh Kothari, Jane Chen, Richard Sidebottom, Victoria Chan, Sarah Yanny, James L Teh

**Affiliations:** ^1^ Stoke Mandeville Hospital, Buckinghamshire Healthcare NHS Trust, Aylesbury, UK; ^2^ Nuffield Orthopaedic Centre, Oxford University Hospitals NHS Trust, Oxford, UK

## Abstract

Pagetic vertebral ankylosis is an uncommon presentation and occurs when Paget's disease is associated with diffuse idiopathic skeletal hyperostosis and ankylosing spondylitis. In these cases, the pagetic trait extends from one vertebra to another by invasion of the intervertebral disc space. Such acquired vertebral ankylosis is extremely uncommon but possible when bony bridging syndesmophytes or osteophytes are present. We describe one such case, where a delayed diagnosis resulted in the most extensive pagetic vertebral ankylosis described in the literature and severe patient morbidity.

## Summary

Paget’s disease (PD) of the bone is a chronic condition of unknown aetiology characterized by a disturbance in bone modelling and remodelling owing to increased osteoblastic and osteoclastic activity. First described in 1876 by James Paget,^1^ it is estimated to affect approximately 3% of the European population, with Britain having the highest prevalence.^2^

The spine is a common site of development of PD, and vertebral involvement will be present in at least 50% of patients with polyostotic disease.^2^ The classic radiological features of PD of the spine include generalized vertebral enlargement with marginal sclerosis, resulting in a “picture frame pattern,” as well as disruption of the normal trabecular architecture and increased density of the neural arch (ivory vertebra).

PD usually starts as a solitary focus within the bone and may spread until the entire bone is affected but does not typically progress to adjacent bones. The soft tissues act as natural barriers to the spread of PD, but when the vertebrae are bridged by syndesmophytes or osteophytes, the disease process can extend to involve the contiguous segments. Pagetic vertebral ankylosis (PVA) is an uncommon presentation and occurs when PD is associated with diffuse idiopathic skeletal hyperostosis (DISH) and ankylosing spondylitis (AS).^3,4^

## Case report

A 73-year-old male was referred to the spinal team with a presentation of becoming “off legs” with progressive difficulty in walking. He had a prior history of vertebral PD, diagnosed 11 years ago, but had reported no symptoms of back pain in the interim.

In the recent months, he had started developing paraesthesia in both lower limbs, with progressive leg weakness and difficulty walking.

There was no bowel or bladder involvement. His past medical history included chronic kidney disease, Type II diabetes mellitus and vitamin D deficiency. He was also noted to be human leukocyte antigen B27 positive.

Clinical examination revealed a rigid thoracic kyphosis and spastic paraparesis. Lower limb power was globally reduced to Medical Research Council grade 4/5. Altered sensation to fine touch was demonstrated below the level of T7.

**Figure 1.**
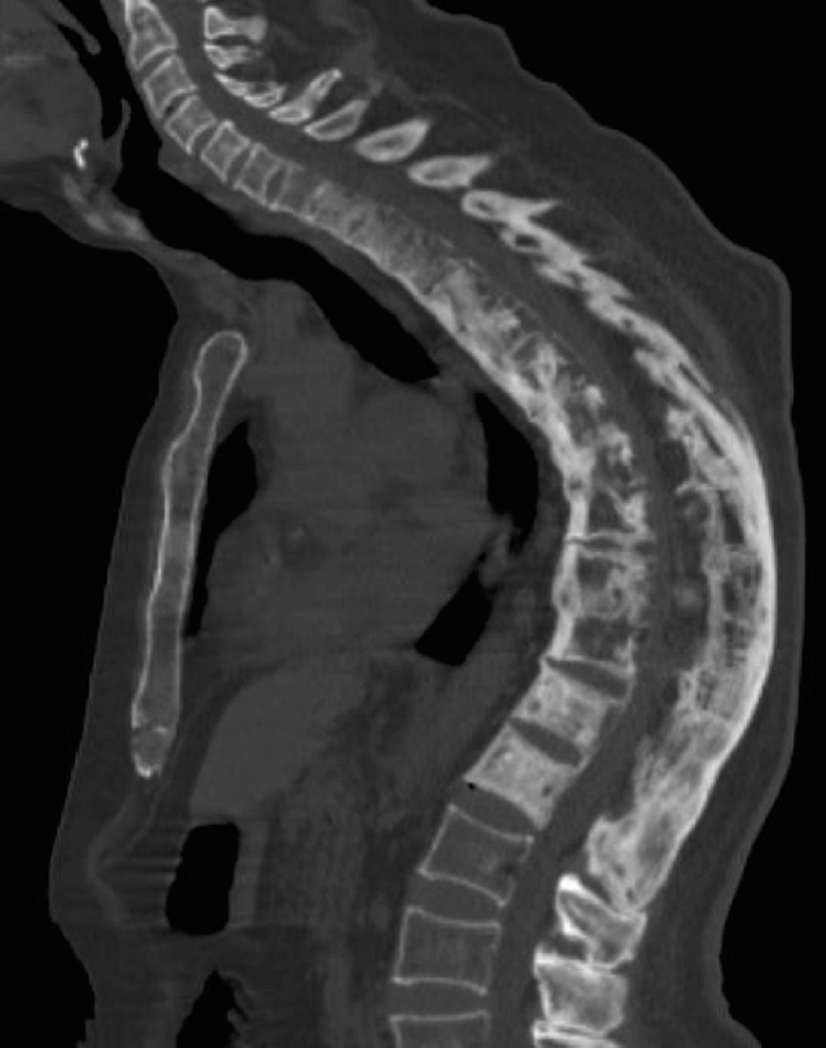
Sagittal CT reformat demonstrating typical features of ankylosing spondylitis, with extensive bridging thoracic syndesmophytes and diffuse ankylosis of the posterior elements. There is coexisting Paget’s disease, with cortical thickening, sclerosis and vertebral squaring. This produces significant kyphotic deformity and narrowing of the central canal.

**Figure 2.**
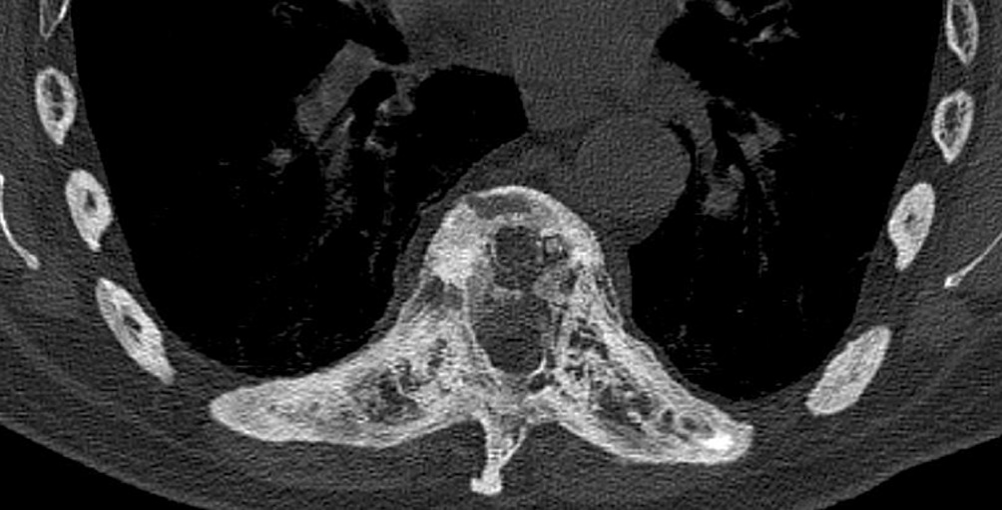
Pagetic expansion of the T10 vertebral body with extension across the fused costovertebral joints. Bony expansion results in marked reduction in the calibre of the vertebral canal.

Subsequent investigations included whole-spine MRI and CT. This demonstrated the features of AS with multilevel syndesmophytes and interspinous ligament calcification. There was cortical thickening, sclerosis and vertebral squaring of T10, consistent with the known history of PD. However, in addition, there was contiguous spread of the pagetic changes across the diffusely ankylosed thoracic segments (Figure 1). The combination of these pathologies produced a marked kyphotic deformity, with extensive bony expansion of the pagetic thoracic spine that resulted in significant central canal stenosis (Figure 2).

**Figure 3.**
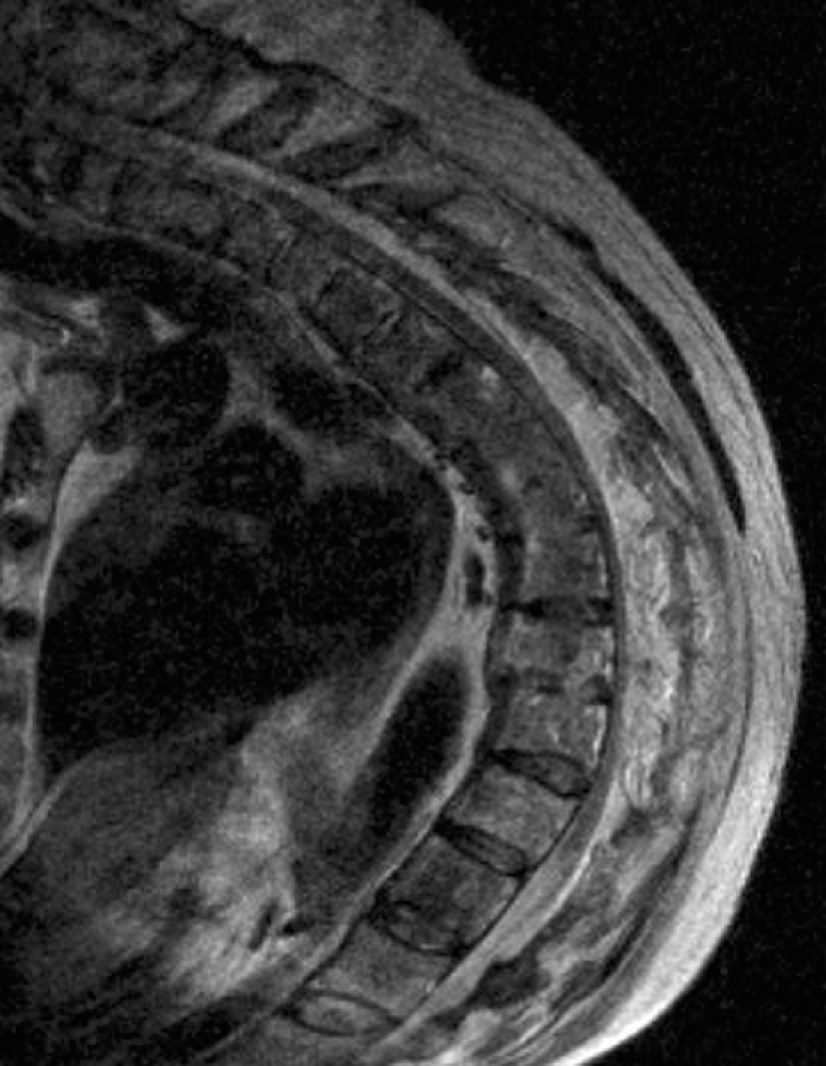
*T*_2_ weighted sagittal MRI of the thoracic spine demonstrating severe central canal stenosis with cord compression and extensive myelopathic change.

**Figure 4.**
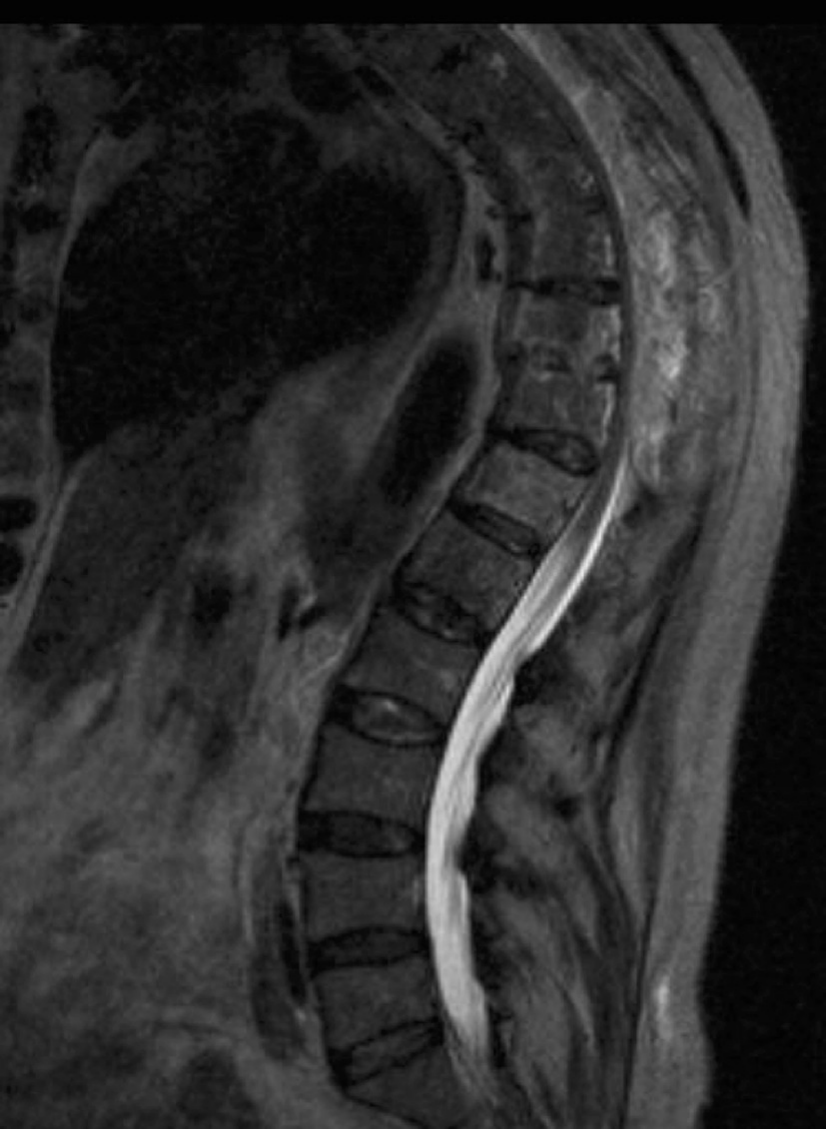
*T*_2_ weighted sagittal MRI of the thoracolumbar spine demonstrating normal appearances of the lumbar segments with a capacious lumbar central canal.

An MRI confirmed the extent of canal stenosis and cord compromise, with intramedullary T2 hyperintensity extending from the cervicothoracic junction to T11 (Figure 3). There was sparing of the lumbar segments, with a normal appearance of the distal cord and conus (Figure 4).

Following discussion at our regional spinal multidisciplinary team, it was decided that surgical decompression would likely result in poor outcome, given the extent and severity of the disease. Multiple infusions of zoledronate were administered, in addition to three courses of calcitonin, in an attempt to delay the progression of the disease. Despite this medical therapy, the patient continued to suffer from progressive myelopathy.

## Discussion

PD, also known as osteitis deformans, is a chronic bone disorder that is common in Western populations, with a prevalence of 5% over the age of 50 years.^5^ The condition is characterized by excessive abnormal bone remodelling. There are three stages classically described as part of a continuous spectrum. The lytic (incipient active) stage is predominated by osteoclastic activity, the mixed active stage is characterized by osteoblastic as well as osteoclastic activity and is followed by the sclerotic/blastic stage (late inactive phase).

PD affecting the spine may lead to cord or nerve root compression through a combination of factors. Myelopathy may be the result of direct compression by abnormal bone growth, ossification of epidural fat and/or sarcomatous change. There may also be ischaemic myelopathy that is related to the local increase in bone turnover, vascular steal phenomenon or compression of a nutrient artery.^6^

In cases of myelopathy due to PD, there is a role for both surgical and medical management. Both calcitonin and bisphosphonates have been used successfully for reversing myelopathic symptoms, presumably by reducing bone turnover.^7^ In cases of severe compressive myelopathy, decompression and stabilization are favoured.

PD can involve the intervertebral disc, resulting in disc degeneration and transductal extension. The incidence of direct intradiscal transgression is about 10.7% and more commonly affects the lumbar spine than the cervical or thoracic segments.^8^ This usually presents with pain; however, it may be asymptomatic. In a study by Lander et al,^8^ 67% of patients with disc involvement presented with pain; however, 22% were asymptomatic. They also report that PD transgressing the intervertebral disc occurs more often in the lumbar spine than in the thoracic and cervical spine. Aggressive pagetic invasion at the discovertebral junction may lead to pagetic tissue replacement of the cartilage endplate and subsequently of the intervertebral disc.^8^ It can then invade the adjoining vertebra across the disc space. This can also occur by direct extension of PD along large pre-existing bridging osteophytes or syndesmophytes^3,8,9^ as in this case.

Intervertebral disc involvement can eventually lead to PVA, an uncommon phenomenon first described by Schmorl^10^ in 1932, having an incidence rate of 4.4%.^8^ PVA is more common in males and affects the thoracic spine in over 50% of cases.^4^

In a cohort of 245 patients, Marcelli et al^4^ found an incidence of 4.4% of PVA due to the coexistence of PD and DISH.^4^ In these cases, a maximum of three ankylosed levels were observed. To the best of our knowledge, no case of PVA involving more than three contiguous vertebra has been described.

AS (also known as Bechterew disease and Marie Strümpell disease) is a seronegative spondyloarthropathy, which results in fusion (ankylosis) of the spine and sacroiliac joints, although involvement is also seen in large and small joints. In our case, the patient had classical radiographic features of AS with bridging syndesmophytes acting as a path of transmission of the pagetic process into the adjacent bone.

Coexisting PD and AS have been previously reported;^3,9,11–15^ however, this is the most extensive case of pagetic vertebral pan-ankylosis described in the literature, with consequent long segment cord compromise. The extent of spinal fusion and deformity, in this case, made surgical intervention impossible. While medical management may reduce bone turnover and vascular demands of the pagetic process, thus reducing the vascular steal phenomenon, it was inadequate to halt the progression of symptoms in this patient. Our case highlights the need for early diagnosis and treatment of pagetic vertebral pan-ankylosis before disease progression limits management options.

## Conclusions

PD with coexisting AS is an uncommon association in which the bridging syndesmophytes provide a path for extension of pagetic bony changes throughout the vertebral column. Pagetic vertebral pan-ankylosis may result in long segment spinal compromise and a poor patient outcome. This should therefore be considered in patients presenting with progressive spinal deformity and rigidity. If there is clinical or radiological suspicion of PD in association with conditions that may result in vertebral body fusion, including AS, DISH or advanced osteoarthritis, prompt investigation, monitoring and early treatment of the underlying PD is advocated.

## Learning points

1. PD with coexisting AS is an uncommon association.
2. In such cases, bridging syndesmophytes may provide a path for extension of the pagetic bony changes.
3. Pagetic vertebral pan-ankylosis may result in long segment spinal compromise and a poor patient outcome.
4. This differential must be considered in patients with known AS presenting with progressive spinal deformity and rigidity, as prompt investigation, monitoring and early treatment of the underlying PD may improve patient outcome.

## Consent

Informed consent has been obtained and is held on record.
